# Imprinted gene detection effectively improves the diagnostic accuracy for papillary thyroid carcinoma

**DOI:** 10.1186/s12885-024-12032-z

**Published:** 2024-03-20

**Authors:** Yanwei Chen, Ming Yin, Yifeng Zhang, Ning Zhou, Shuangshuang Zhao, Hongqing Yin, Jun Shao, Xin Min, Baoding Chen

**Affiliations:** 1 Department of Medical Ultrasound, Affiliated Hospital of Jiangsu University, 212000 Zhenjiang, Jiangsu China; 2 Department of Medical Ultrasound, The Affiliated Taizhou People’s Hospital of Nanjing Medical University , 225300 Taizhou, Jiangsu China; 3 Department of Medical Ultrasound, Shanghai Tenth People’s Hospital, Ultrasound Research and Education Institute, Shanghai Engineering Research Center of Ultrasound Diagnosis and Treatment, School of Medicine, Tongji University, 200072 Shanghai, China; 4 Lisen Imprinting Diagnostics, Inc., 214135 Wuxi, Jiangsu China; 5 Department of Medical Ultrasound, The First People’s Hospital of Kunshan, 215300 Kunshan, Jiangsu China

**Keywords:** Papillary thyroid carcinoma, Prediction model, Imprinted gene detection, Nomogram, Ultrasound features

## Abstract

**Background:**

Papillary thyroid carcinoma (PTC) is the most frequent histological type of thyroid carcinoma. Although an increasing number of diagnostic methods have recently been developed, the diagnosis of a few nodules is still unsatisfactory. Therefore, the present study aimed to develop and validate a comprehensive prediction model to optimize the diagnosis of PTC.

**Methods:**

A total of 152 thyroid nodules that were evaluated by postoperative pathological examination were included in the development and validation cohorts recruited from two centres between August 2019 and February 2022. Patient data, including general information, cytopathology, imprinted gene detection, and ultrasound features, were obtained to establish a prediction model for PTC. Multivariate logistic regression analysis with a bidirectional elimination approach was performed to identify the predictors and develop the model.

**Results:**

A comprehensive prediction model with predictors, such as component, microcalcification, imprinted gene detection, and cytopathology, was developed. The area under the curve (AUC), sensitivity, specificity, and accuracy of the developed model were 0.98, 97.0%, 89.5%, and 94.4%, respectively. The prediction model also showed satisfactory performance in both internal and external validations. Moreover, the novel method (imprinted gene detection) was demonstrated to play a role in improving the diagnosis of PTC.

**Conclusion:**

The present study developed and validated a comprehensive prediction model for PTC, and a visualized nomogram based on the prediction model was provided for clinical application. The prediction model with imprinted gene detection effectively improves the diagnosis of PTCs that are undetermined by the current means.

**Supplementary Information:**

The online version contains supplementary material available at 10.1186/s12885-024-12032-z.

## Introduction

Thyroid carcinoma (TC) is the most common malignancy in the endocrine system, which has been ranked fifth among malignancies in women [1, 2]. Through widespread application of high-resolution ultrasound, the detection rate of TC has significantly increased [3]. Papillary thyroid carcinoma (PTC), which is the most common variety of differentiated TC, has a good prognosis in most instances [4]. However, a few aggressive histologic variants of PTC may develop recurrence and metastasis, remaining the primary threat to the lives of PTC patients [5, 6]. Currently, the most frequently used diagnostic protocol is to first determine whether fine-needle aspiration (FNA) biopsy for cytological diagnosis is needed based on the sonographic appearance of the nodules prior to performing supplemental testing with several molecular markers in accordance with the cytopathological results [7]. Disappointedly, FNA still remains indeterminate cytological categories, while molecular testing is limited to geographical variation [8]. Therefore, for thyroid nodules that cannot be accurately diagnosed with existing clinical technologies, the common practice is to undertake surgical interventions, such as lobectomy or total thyroidectomy, which contribute to overdiagnosis and overtreatment in some cases, causing patients to take unnecessary risks [9–12]. In view of this, accurately distinguishing benign and malignant thyroid nodules has become the most crucial question that needs to be answered in the initial evaluation, and a novel method urgently needs to be developed [13].

Genomic imprinting is an epigenetic mechanism that plays a critical role in human development and diseases [14]. Under normal conditions, imprinted genes are only expressed from the maternal or paternal allele in post-embryonic somatic cells because one copy is repressed by epigenetic markers, such as DNA methylation and histone acetylation. However, in some cases, imprinted genes may be abnormally activated through demethylation, resulting in expression from both alleles. This phenomenon has been identified as loss of imprinting (LOI), which occurs in a variety of human malignancies [15]. Based on this finding, Shen et al. developed a novel approach named Quantitative Chromogenic Imprinted Gene In Situ Hybridization (QCIGISH) to quantify and visualize the allele-specific expression of imprinted genes in cell nuclei [16]. Conceptually different from other molecular tests which identify genetic mutations, QCIGISH detects epigenetic imprinting alterations through LOI which represent the earliest visible change in human cancers. LOI can occur early in the process of cancer development preceding genetic mutations, thus providing a potential window for early cancer detection at its more curable stage. Later, this method was utilized by Xu et al. in their clinical research, which indicated the excellent capability to discriminate malignant from benign thyroid nodules [17]. To improve the assessment and management of PTC, this new method was used in the present study.

Herein, the present study was focused on patients recruited from two independent centres to develop and validate a comprehensive prediction model for PTC using patients’ general information, imprinted gene detection, cytopathology, and ultrasound features. The present findings are expected to further improve the clinical treatment effects and prognosis of PTC patients.

## Materials and methods

The development and validation of the prediction model is reported in light of the TRIPOD checklist [18].

### Study design and patients

The retrospective study of two independent centres was conducted after its approval by the Ethics Committee of the Affiliated Hospital of Jiangsu University, and informed consent was obtained from all patients. The development cohort included consecutive patients from the Affiliated Hospital of Jiangsu University, and the validation cohort included patients from The Affiliated Taizhou People’s Hospital of Nanjing Medical University. The patients were admitted to the two centres between August 2019 and February 2022. All patients underwent ultrasound examination, FNA biopsy, imprinted gene detection, and thyroid surgery. Cases with complete data were included in the study, except for patients meeting the following criteria: (a) history of radiation exposure in adolescence or a family history of TC; (b) previous subtotal thyroidectomy for TC; (c) postoperative histopathology showing other types of TC apart from PTC; (d) cytopathology specimen that was not satisfactory or failed to be diagnosed; (e) imprinted gene detection that was unable to be diagnosed; and (f) abnormal thyroid function test results.

### Model variables and outcome

The candidate model variables included the patients’ general information, ultrasound features, cytopathology and imprinted gene detection. The general information comprised the patients’ age and sex. In accordance with the relevant guidelines and literature, the following variables were included in the ultrasound features: maximum diameter, component, echogenicity, margin, shape, location, microcalcification and blood flow of the nodule [19–21]. MylabTwice (Esaote, Genova, Italia) and LOGIQ E8 (General Electric, Boston, United States of America) were utilized to perform ultrasound examination, and the ultrasonic graphics were reviewed by two experienced radiologists at the respective centres to attain the required ultrasound features. Adler grade classifications were conducted to assess blood flow of the nodules, and the results were indicated by 4 grades as follows: (0) no blood flow signal; [1] one or two spot-like blood flow signals; [2] two or three spot-like or short strip blood flow signals; and [3] sheet-like, striped, or dendritic blood flow signals [22]. The reported cytopathology results referred to the following Bethesda System: (I) Nondiagnostic; (II) Benign; (III) Atypia of undetermined significance; (IV) Follicular neoplasm (V) Suspicious for malignancy; (VI) Malignant [23]. In the present study, the nodules with Category II were classified to the benign group, Category III, IV and V to the indeterminate group and Category VI to the malignant group [23, 24]. The punctured cytology specimens were sent to a professional testing institution (Lisen Imprinting Diagnostics, Wuxi, China) for imprinted gene detection, and the results were divided into 5 grades (Grades 0–IV) as follows: Grades 0 and I were considered negative, while Grades II, III, and IV were considered positive [17].

The outcome of interest was PTC confirmed by postoperative histopathology. Formalin fixation and paraffin embedding (FFPE) nodule specimens were obtained after the operation, and the pathological results were confirmed by two experienced pathologists independently according to the 4th edition of the World Health Organization (WHO) classification of head and neck tumours [25]. When the results were inconsistent, another senior pathologist was asked to assess the findings and determine the final result. During the entire process, the pathologists were unaware of the patients’ other examination results.

### Imprinted gene detection

The imprinted gene detection was based on QCIGISH technology [16]. The FNA specimens were fixed in 10% formalin neutral buffer immediately after sampling, mounted on positively charged slides, and dried overnight. The sample slides were pretreated following the RNAscope sample preparation procedures [26]. QCIGISH was performed using probes targeting the noncoding intronic regions of nascent RNAs from the *SNRPN* and *HM13* imprinted genes [17]. After signal amplification and haematoxylin staining, the slides were scanned under a 400× bright field microscope. The number of nuclei containing no signal (no expression = N_0_), one signal (single-allelic expression = N_1_), two signals (biallelic expression = N_2_), and more than two signals (multiallelic expression = N_2plus_) were counted from at least 1,200 nuclei for each sample per gene. The biallelic expression (BAE), multiallelic expression (MAE), and total expression (TE) were then calculated according to the following equations:

$$ \text{B}\text{A}\text{E}=\frac{{N}_{2}}{{N}_{1}{+N}_{2}{+N}_{2plus}}\times 100\%$$

$$ \text{M}\text{A}\text{E}=\frac{{N}_{2plus}}{{N}_{1}{+N}_{2}{+N}_{2plus}}\times 100\%$$

$$ \text{T}\text{E}=\frac{{N}_{1}{+N}_{2}{+N}_{2plus}}{{N}_{0}{+N}_{1}{+N}_{2}{+N}_{2plus}}\times 100\%$$

The QCIGISH grades for each case were calculated according to the previously reported diagnostic model, and detailed information is provided in the Supplementary Fig. 1, Additional file 1 [17].

**Fig. 1:**
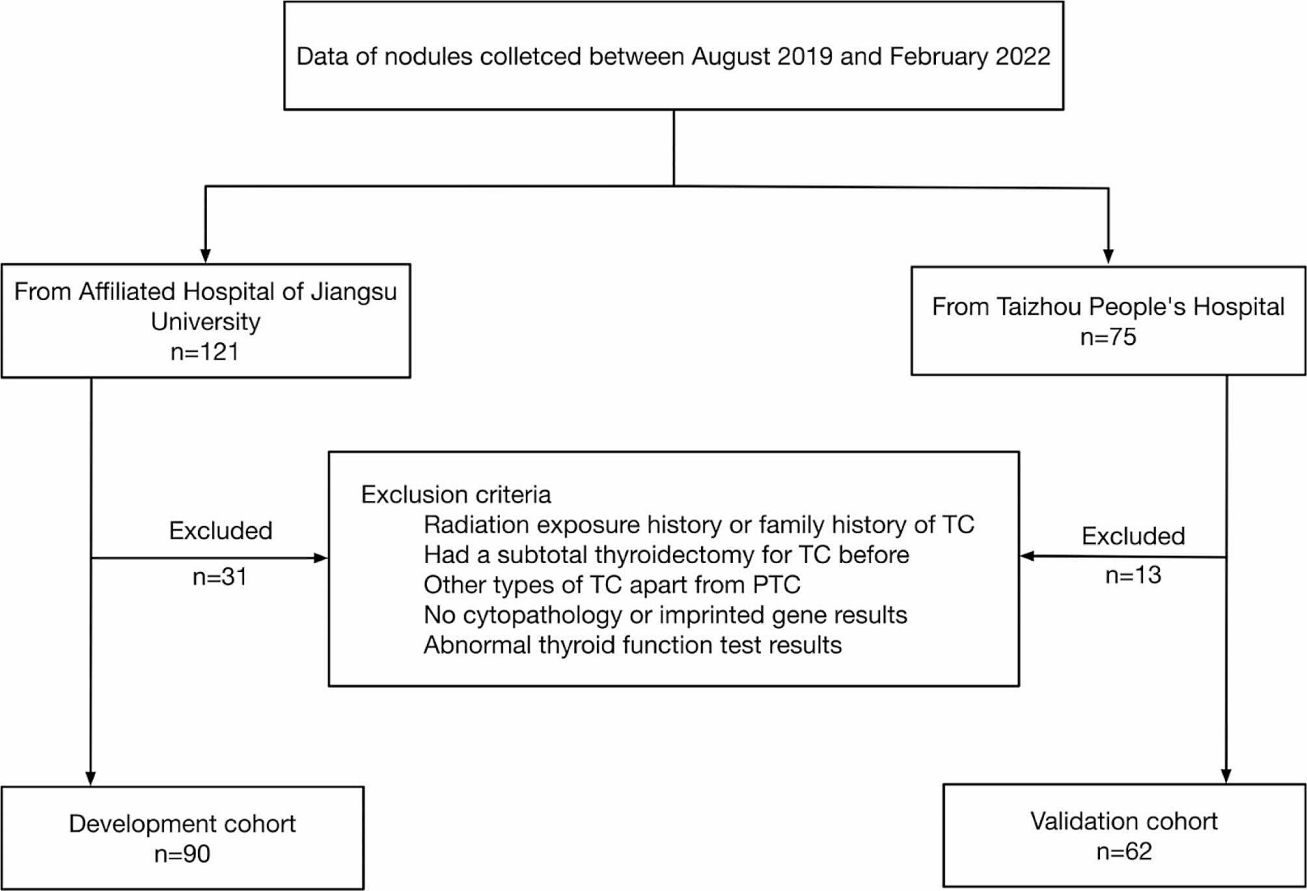
The flowchart of the nodules selected into the development and validation cohorts from two centers

### Model development

To develop the prediction model, age and maximum diameter were considered continuous variables, while the other variables were considered categorical variables. First, by performing univariate analysis, variables that were significantly different (*P* < 0.05) between the benign and malignant groups were considered potential predictors. Next, the model was established through multivariate logistic regression analysis with a bidirectional elimination approach. When the model had the minimal Akaike information criterion (AIC) value with the best goodness of fit, the variables with *P* < 0.05 were finally selected into the Model [27]. Cook’s distance and variance inflation factor (VIF) were used to detect abnormal data and multicollinearity of the variables, respectively. The performance of the model was presented using the receiver operating characteristic (ROC) curve and area under the curve (AUC).

### Model validation

The final model was validated using both internal and external validation. The enhanced bootstrap method (number of repetitions = 100) was utilized to internally validate with optimism and mean absolute error. To validate the results externally, the AUC, Brier score, calibration intercept, calibration curve coupled with calibration, and discrimination slopes were plotted or calculated.

Furthermore, we evaluated the robustness of the model with respect to the changes in the external validation data. Firstly, the R package simFrame was used to artificially create 10% missing values through the method of missing completely at random (MCAR) [28]. Subsequently, multiple imputation was performed on the missing data using the random forest method through the R package mice [29]. Finally, the new dataset was evaluated by the model to calculate evaluation metrics.

### Model comparison and model visualization

To compare the models with and without variable imprinted gene detection, we compared their AUC through the DeLong test, and we performed model fitting using the likelihood ratio (LR) test [30]. The clinical net benefit was evaluated by the decision curve analysis (DCA) curve [31]. In addition, the diagnostic ability was compared with net reclassification improvement (NRI) and integrated discrimination improvement (IDI) [32]. Finally, a nomogram and web-based dynamic nomogram were developed to visualize the prediction model.

### Statistical analysis

All statistical analyses were performed with SPSS 26.0 (International Business Machines Corporation, New York, United States of America) and R version 4.0.2 (The R Foundation for Statistical Computing, Vienna, Austria).

## Results

### Patient characteristics

In accordance with the exclusion criteria, 44 patients were excluded from the development and validation cohorts (Fig. 1). The development cohort included 90 nodules, while the validation cohort included 62 nodules. In the development cohort, nodules were divided into benign (*n* = 57) and malignant (*n* = 33) groups based on postoperative histopathological results. The median age was 47.0 (i.q.r 40.5–56.0) years in the benign group and 48.0 (i.q.r 35.0–53.5) years in the malignant group. Female patients made up the majority in both the benign (77.2%) and malignant (69.7%) groups. The internal echogenicity of the nodules in the two groups was dominated by hypoechoicity. In addition, nodules commonly occurred in the middle lobe of the thyroid, accounting for 56.1% and 63.6% of nodules in the benign and malignant groups, respectively. Further baseline information for the two cohorts is shown in Table 1.

**Table 1:** The baseline of patients in development and validation cohort

	Development			Validation		
	Benign (n = 57)	Malignant (n = 33)	*P*	Benign (n = 26)	Malignant (n = 36)	*P*
Age, y	47.0 (40.5–56.0)	48.0 (35.0–53.5)	0.58	45.0 (32.8–51.8)	43.5 (35.3–52.5)	0.87
Sex ratio (M: F)	1 : 3.4	1 : 2.3	0.43	1 : 5.5	1 : 6.2	0.75
Maximum diameter, mm	13.0 (8.5–23.5)	13.0 (9.0–16.5)	0.42	16.0 (10.0–25.5)	12.0 (8.3–15.0)	< 0.01
Component			0.04			0.05
*Solid*	44 (77.2)	31 (93.9)		18 (69.2)	32 (88.9)	
*Partially cystic*	13 (22.8)	2 (6.1)		8 (30.8)	4 (11.1)	
Echogenicity			0.12			0.92
*Markedly hypoechoic*	10 (17.5)	11 (33.3)		6 (23.1)	7 (19.4)	
*Hypoechoic*	29 (50.9)	17 (51.5)		13 (50.0)	20 (55.6)	
*Iso-echoic*	14 (24.6)	2 (6.1)		5 (19.3)	7 (19.4)	
*Hyperechoic*	1 (1.8)	0 (0)		1 (3.8)	0 (0)	
*Mix-echoic*	3 (5.3)	3 (9.1)		1 (3.8)	2 (5.6)	
Margin			0.02			< 0.01
*Microlobulated*	8 (14.0)	13 (39.4)		2 (7.7)	18 (50.0)	
*Irregular*	25 (43.9)	12 (36.4)		8 (30.8)	12 (33.3)	
*Well circumscribed*	24 (42.1)	8 (24.2)		16 (61.5)	6 (16.7)	
Shape			0.67			< 0.01
*Wider than taller*	47 (82.5)	7 (21.2)		19 (73.1)	8 (22.2)	
*Taller than wider*	10 (17.5)	26 (78.8)		7 (26.9)	28 (77.8)	
Location			0.67			0.28
*Upper*	8 (14.0)	6 (18.2)		8 (30.8)	14 (38.9)	
*Middle*	32 (56.1)	21 (63.6)		11 (42.3)	16 (44.4)	
*Lower*	12 (21.1)	4 (12.1)		7 (26.9)	4 (11.1)	
*Isthmus*	5 (8.8)	2 (6.1)		0 (0)	2 (5.6)	
Microcalcification			< 0.01			< 0.01
*Yes*	5 (8.8)	19 (57.6)		2 (7.7)	30 (83.3)	
*No*	52 (91.2)	14 (42.4)		24 (92.3)	6 (16.7)	
Blood flow			0.60			0.63
*Grade 0*	21 (36.8)	12 (36.4)		13 (50.0)	15 (41.7)	
*Grade 1*	26 (45.6)	18 (54.5)		6 (23.1)	13 (36.1)	
*Grade 2*	4 (7.0)	2 (6.1)		4 (15.4)	6 (16.6)	
*Grade 3*	6 (10.5)	1 (3.0)		3 (11.5)	2 (5.6)	
Imprinted gene detection			< 0.01			< 0.01
*Grade 0*	26 (45.6)	0 (0)		16 (61.5)	0 (0)	
*Grade I*	22 (38.6)	1 (3.0)		5 (19.2)	4 (11.1)	
*Grade II*	5 (8.8)	10(30.3)		3 (11.5)	12 (33.3)	
*Grade III*	4 (7.0)	14 (42.4)		2 (7.8)	17 (47.3)	
Grade IV	0 (0)	8 (24.3)		0 (0)	3 (8.3)	
Cytopathology			< 0.01			< 0.01
*Benign (Category II)*	20 (35.0)	1 (3.0)		17 (65.4)	0 (0)	
*AUS (Category III)*	35 (61.4)	9 (27.3)		5 (19.2)	3 (8.3)	
*FN (Category IV)*	1 (1.8)	4 (12.1)		4 (15.4)	4 (11.1)	
*SUS (Category V)*	1 (1.8)	7 (21.2)		0 (0)	24 (66.7)	
*Malignant (Category VI)*	0 (0)	12 (36.4)		0 (0)	5 (13.9)	

*Note*: Values are presented as n (%) or median (i.q.r)

*Abbreviations*: AUS, atypia of undetermined significance; FN, follicular neoplasm; SUS, suspicious for malignancy

### Model development

**Fig. 2:**
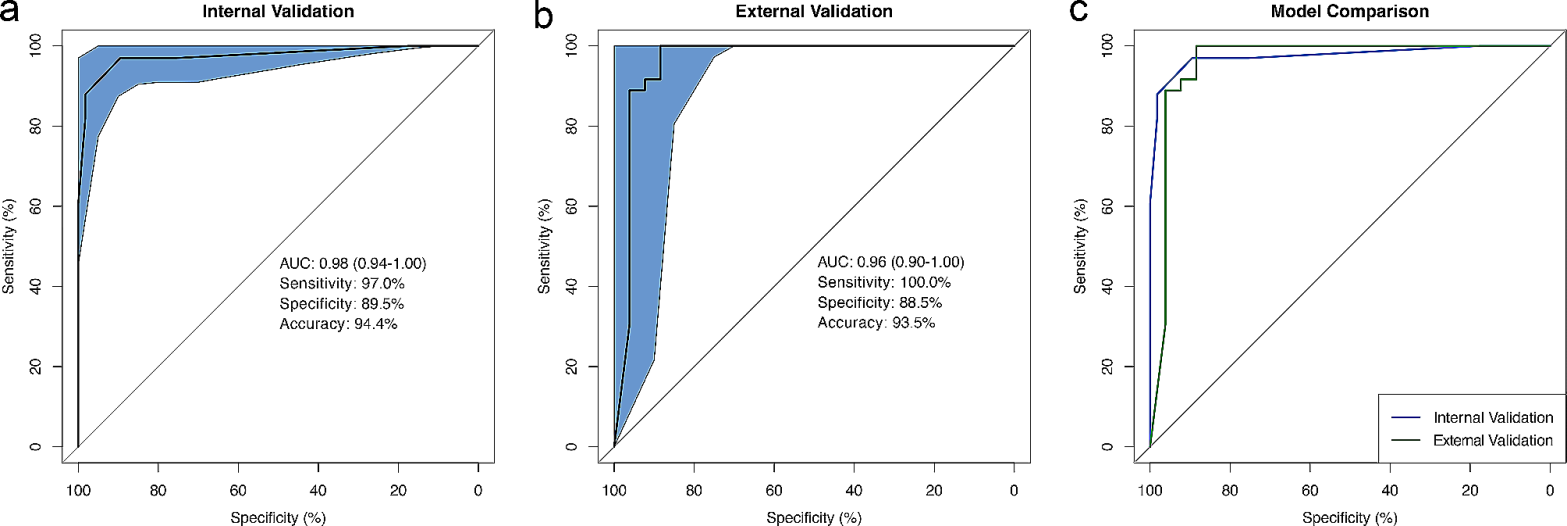
The receiver operating characteristic (ROC) curves of the prediction model in the development (a) and external validation cohorts (b). (c) The comparison of ROC curves between the prediction model in the development and external validation cohorts

The component, margin, microcalcification, cytopathology, and imprinted gene detection were identified as potential predictors of PTC (*P* < 0.05) via univariate analysis. Next, multivariable logistic regression analysis demonstrated that four variables, namely, component (*Z*: 2.71, *P* < 0.01), microcalcification (*Z*: 2.35, *P* = 0.02), cytopathology (*Z*: 2.54, *P* = 0.01), and imprinted gene detection (*Z*: 3.50, *P* < 0.01), showed statistical significance and entered the final model (Table 2). The AIC of the model was 41.36, while the Cook’s distances calculated to discriminate the influential cases showed that the largest one was smaller than 0.40. Consequently, the equation of the final prediction model for PTC was *Y* = -10.62 + 5.52 (component) + 3.23 (microcalcification) + 2.02 (cytopathology) + 4.84 (imprinted gene detection). The VIFs of component, microcalcification, cytopathology, and imprinted gene detection were 2.25, 1.56, 1.76, and 1.35, respectively. The AUC of the ROC was 0.98 (95% CI: 0.94–1.00) (Fig. 2a). The sensitivity, specificity, and accuracy of the model were 97.0%, 89.5%, and 94.4%, respectively.

**Table 2:** The multivariable logistic regression results of candidate variables for the model

Variables	β	Z	P	95% CI
Component Microcalcification Cytopathology Imprinted gene detection	5.52 3.23 2.02 4.84	2.71 2.35 2.54 3.50	< 0.01 0.02 0.01 < 0.01	1.52–9.52 0.54–5.93 0.46–3.57 2.13–7.55

*Abbreviations* β, regression coefficient; CI, confident interval

### Model validation

**Fig. 3:**
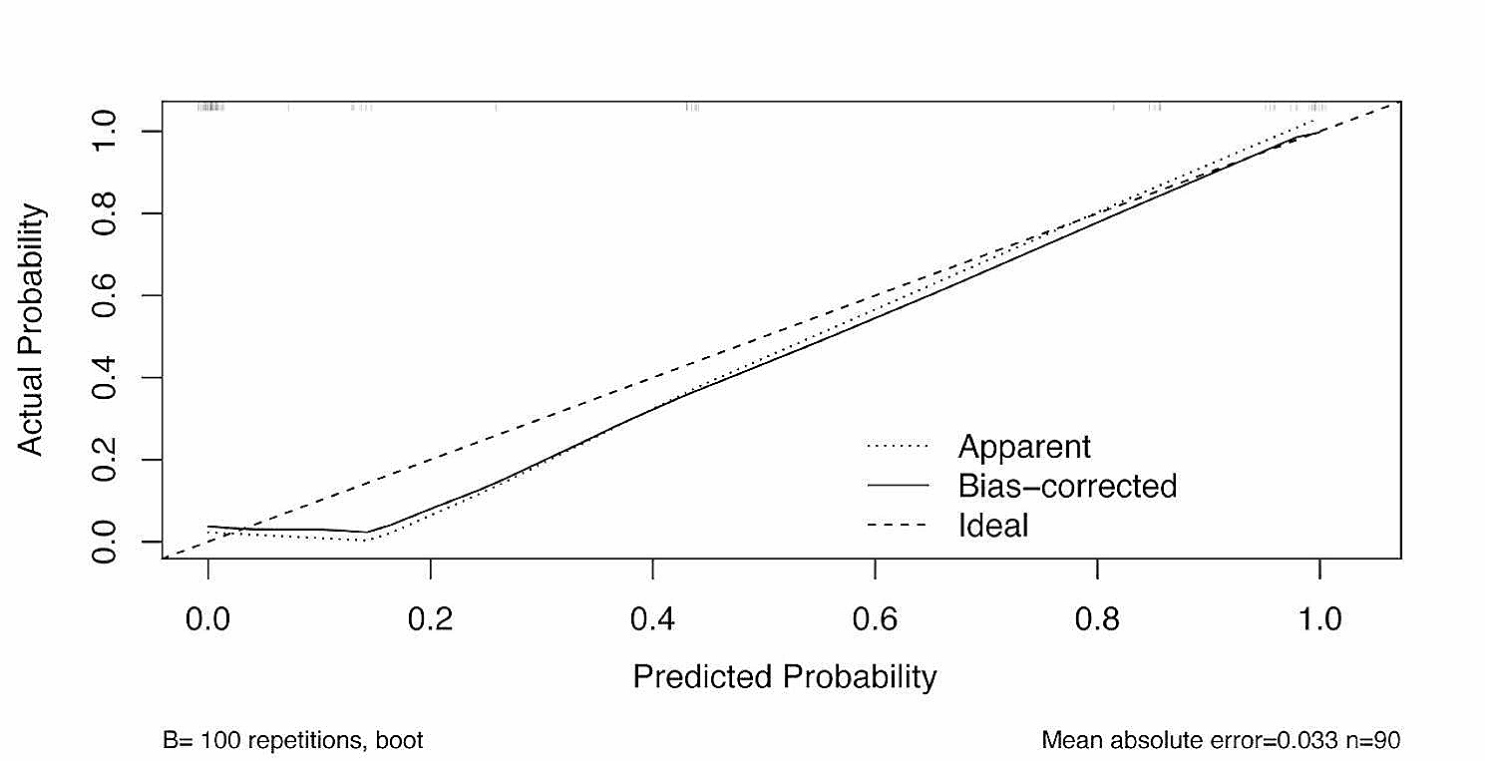
The calibration curve of the prediction model via internal validation with enhanced bootstrap method

The model was subjected to both internal and external validation. Through internal validation with enhanced bootstrapping, the calibration curve was plotted (Fig. 3) to check the internal validity, and the predicted PTC was in agreement with the actual observation after internal validation, thereby indicating that the model had good calibration. The mean absolute error was 0.033, while the mean squared error was 0.002. The corrected *C*-statistic was 0.98, with a mean optimism of 0.01.

**Fig. 4:**
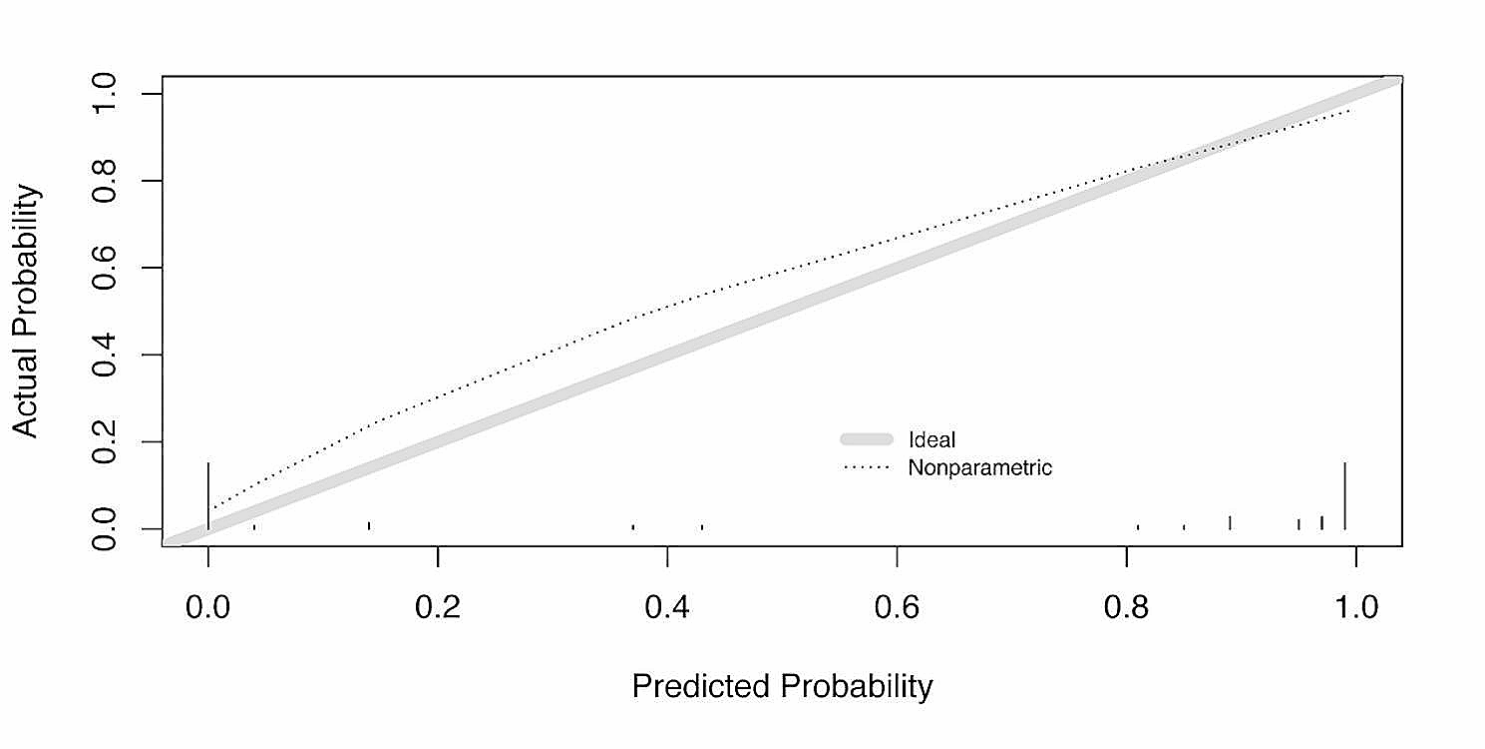
The calibration plot of the prediction model through external validation

External validation was conducted using a validation cohort comprising 62 nodules, and an ROC curve was generated (Fig. 2b and c). In applying the model to the data derived from the validation cohorts, the AUC was 0.96 (95% CI: 0.90–1.00), and the Brier score was 0.06. The sensitivity and specificity values were 100.0% and 88.5%, respectively. The calibration intercept and calibration slope were 0.40 and 0.63, respectively, which indicated good overall calibration (Fig. 4) [33]. In addition to the AUC, the discrimination slope, which was 0.82, was used to assess the discrimination of the model.

Sensitivity analysis was conducted to verify the robustness of the model. 10% of the external validation data were randomly missing and imputed by the MICE algorithm. The sensitivity and specificity values were 100.0% and 80.8%, respectively. The AUC, Brier score, calibration intercept and calibration slope of the external validation were 0.95, 0.10, 0.36 and 0.57, respectively after re-evaluating the model. The ROC curves and calibration curves plotted are shown in the Supplementary Fig. 2, Additional file 1.

### Model comparison

**Fig. 5:**
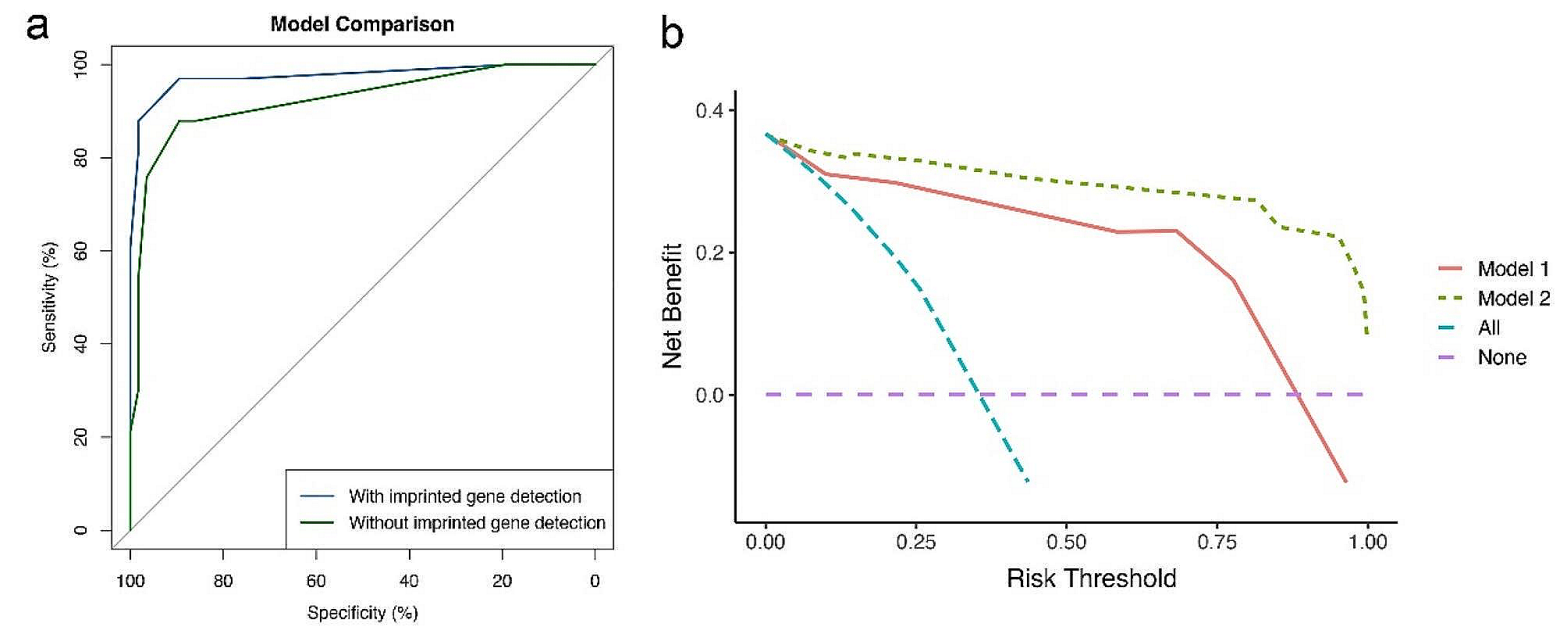
(**a**) The comparison of ROC curves between the prediction model with the predictor namely imprinted gene detection and without it. (**b**) The decision curve analysis (DCA) curves showing the net clinical benefit of the models. Model 1 represents the model without the predictor namely imprinted gene detection and Model 2 with it. The curve All and None represent the situation that all cases are intervened and none of them are intervened, respectively

In the present study, imprinted gene detection was the method of choice due to its novelty and recent clinical trials. To study the role of imprinted gene detection in the prediction model, we compared the difference in statistics between the model with and without it. First, we compared the ROC curves of the two models and calculated their AUC values (Fig. 5a). The AUC of the model with imprinted gene detection was 0.98, while that of the model without imprinted gene detection was 0.92. Although there was little difference in the AUC values between the two models, the DeLong test, which is based on variance and covariance, showed *Z* = -2.13 (*P* = 0.03), demonstrating that the difference between the two models was significant. Thus, there were advantages in using a model with a predictor of imprinted gene detection than without it. Next, to identify the improvements of models in risk predictions, NRI and IDI were performed (NRI [Categorical]: 0.09, 95% CI: -0.06–0.24, *P* = 0.22; NRI [Continuous]: 1.69, 95% CI: 1.49–1.90, *P* < 0.01; IDI: 0.18, 95% CI: 0.11–0.25, *P* < 0.01). Furthermore, the LR test was conducted to compare the model fitting of the two models (L.R. Chisq: 2.42; *P* < 0.01), indicating that the model with imprinted gene detection was better than that without it. Finally, DCA was applied to compare the clinical application and benefits between the two models, further confirming that the model with imprinted gene detection showed favourable effects for PTC patients (Fig. 5b).

### Model visualization

**Fig. 6:**
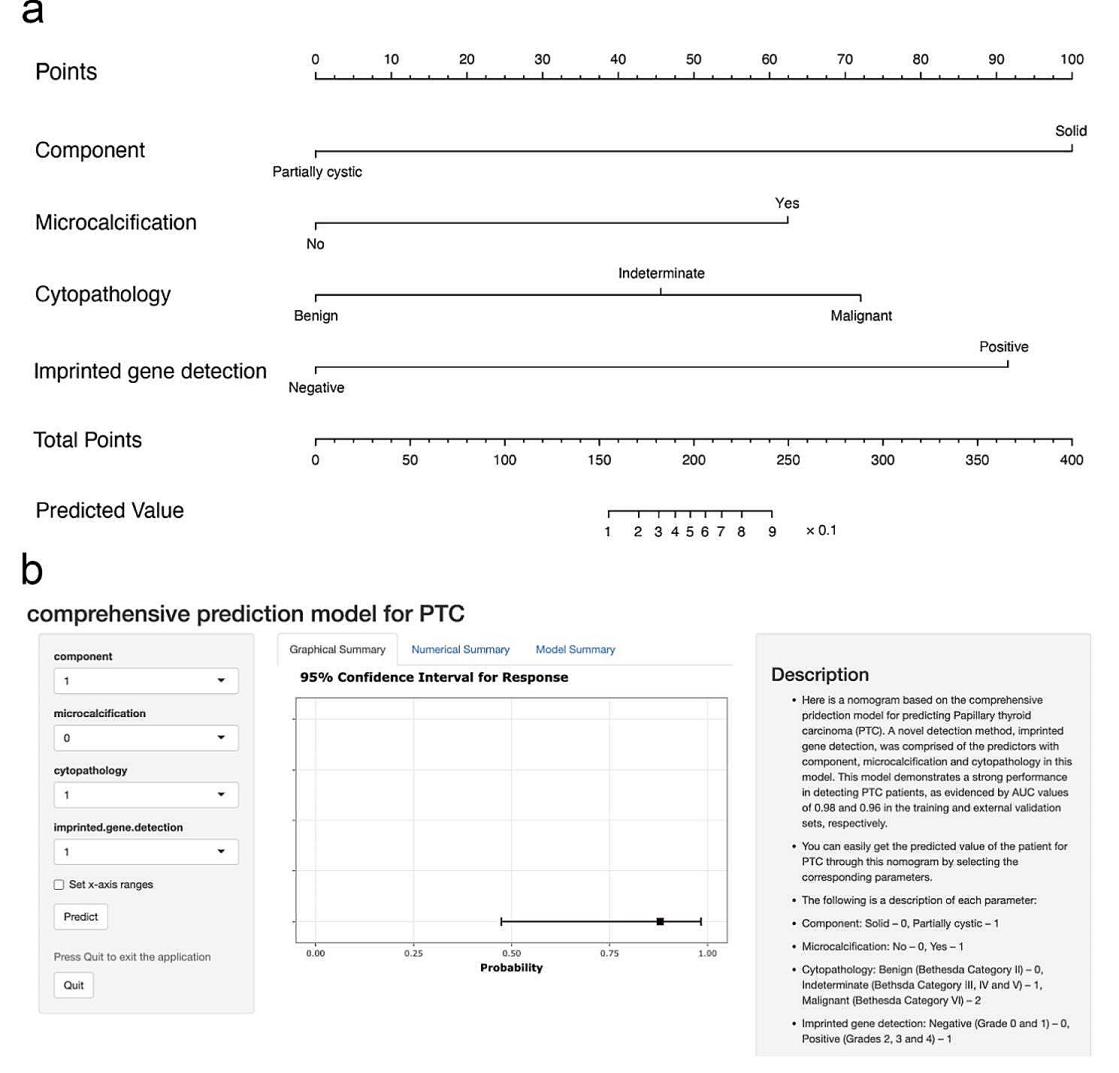
The static (**a**) and online dynamic (**b**) nomogram based on the comprehensive prediction model for predicting papillary thyroid carcinoma

To avoid using the complicated formula and better apply it to clinical settings, we combined all the variables in the final prediction model to construct a nomogram for predicting PTC (Fig. 6a). Summing up the points of the four variables, the acquired total points effortlessly yielded the corresponding predicted values. Moreover, an online dynamic nomogram (Fig. 6b) was built to further simplify the manipulations (https://cywujs.shinyapps.io/modelPTC/).

## Discussion

In the present study, we retrospectively analysed the general information, ultrasound features, cytopathology, and imprinted gene detection in a total of 152 nodules, and we established a comprehensive prediction model for PTC. The model had satisfactory performance in both internal and external validation, demonstrating that imprinted gene detection has advantages in increasing diagnostic efficiency. Based on the prediction model, a nomogram was provided for clinical application.

Ultrasound is recognized as the first priority and plays an important role in the diagnosis of thyroid nodules [20]. In the present study, eight ultrasound features that may be related to PTC were used as candidate variables for the model [19, 34, 35]. The present results showed that the component was the most significant variable among the ultrasound features. As reported, approximately 88% of PTCs have solid components, while carcinomas with marked cystic changes are relatively rare, accounting for only 6% of lesions [36, 37]. In addition, a recent study further demonstrated that the sensitivity of solid components is the highest of all ultrasound features [38]. Microcalcification was also included as a predictor in the model. Previous studies have reported that microcalcification is closely related to PTC based on coloured Doppler images combined with contrast-enhanced ultrasonography (CEUS), showing that microcalcification is a strong predictor for PTC [39, 40]. However, various scholars have also proposed that CEUS has difficulty in determining whether the tiny hyperechoic foci derived from microcalcifications or colloid crystals sometimes lead to biases in predictions of the model, thus requiring constant observation in subsequent work [41]. Moreover, it is worth noting that nodules with irregular margins have been reported to be associated with malignancy [20]. In the present study, the margin of the nodules was significantly different (*P* = 0.02) between the two groups, but multivariate logistic regression analysis indicated that the margin did not show statistical significance (*P* = 0.75), which may be because the irregular margin of a few small nodules could not be clearly displayed on the ultrasound images.

Ultrasound-guided FNA biopsy is the most recommended method for nodules that are difficult to diagnose under ultrasound emanations, especially for nodules with maximum dimensions equal to or more than 1 cm and high suspicion ultrasound features [19]. According to different guidelines, the sensitivity and specificity of diagnostic performance for FNA range from 70.9 to 94.5% and 26.4–62.4%, respectively [42]. In the majority of hospitals, cytopathological examination is regarded as a crucial factor in determining the surgical strategy, and the result is sufficient for making accurate clinical management decisions in most situations [43]. Nevertheless, Category III and IV nodules, which account for approximately 20–30% of the results, are indeterminate, and the rates of malignancy range from 20 to 30% and 25–40%, respectively [19]. The uncertainty in cytopathology greatly increases the confusion for both doctors and patients, resulting in repeat FNA or unnecessary surgery [44].

It has been reported that up to 30% of cases tested in cytopathology lack the morphological features needed to provide a definitive classification. Thus, molecular tests have been developed to help diagnose these uncertain cases and have been recommended by the ATA guidelines [19, 45]. The analysis of the *BRAF* mutation has emerged as a significant advancement in the molecular diagnosis of thyroid carcinoma in recent years, garnering extensive research attention [46]. In addition, a variety of biomarkers have been used clinically, such as *RAS* mutation, *RET/PTC* rearrangement, and *PAX8-PPARγ* rearrangement [47]. Considerable progress has been made in the continuous development of molecular testing platforms, including Afirma Genomic Sequencing Classifier (GSC) and ThyroSeq v3, which are currently the two main thyroid molecular classifiers with proven clinical efficacy, but both have some limitations, such as low specificity, low positive predictive value, or high price [48].

During the past decade, there has been increasing interest in the relationship between epigenetics and diseases. The rapid development of epigenetics in the 21st century has provided researchers with new ideas for the occurrence and development of diseases [49]. Epigenetic changes, especially variations in epigenetic markers, have been considered to play a role in the diagnosis and prognosis of up to 80% of carcinomas [50]. Genomic imprinting, which is one of the epigenetic mechanisms and the biallelic expression caused by LOI, is ubiquitous in different types of carcinomas [51]. Based on this phenomenon, the QCIGISH method was developed to visualize and quantitatively analyse noncoding RNA allelic expression of three imprinted genes with the purpose of early diagnosis of ten types of carcinomas, including PTC. The sensitivity and specificity of QCIGISH for predicting TC have been reported to be 91% and 86%, respectively [16]. In a recent clinical study, imprinted gene detection using an improved gene combination of *SNRPN* and *HM13* demonstrated a sensitivity of 100% [17]. To address false-positives in imprinted gene detection, a comprehensive model combining imprinted gene detection with other available clinical detections may be helpful. The present prediction model is the first to utilize imprinted gene detection combined with traditional predictors for predicting PTC, with a sensitivity of 97.0% and specificity of 89.5%. The comparison between the models with and without imprinted gene detection further confirmed the meaningful role that imprinted gene detection has in predicting PTC. The results revealed that the model with imprinted gene detection not only had a higher AUC but also had the ability to include malignant nodules omitted as benign and exclude benign nodules misdiagnosed as malignant by the model without it. Furthermore, the results indicated that the model with imprinted gene detection had a better model fitting. Various studies have indicated that the addition of imprinted gene detection could further diagnose nodules that were originally indeterminate under cytopathology and ultrasound features.

There were several limitations in the present study. First, the number of nodules was not sufficient, which may have influenced the predictive effect of the model. Second, the present study is only applicable to the diagnosis of patients with suspected PTC, while it remains unknown whether it is effective in the diagnosis of other types of TC. Last, the patients recruited for developing and validating the model were all from one province, and subsequent research is needed to determine whether geographical variations affect the accuracy of the model’s prediction.

## Conclusion

In conclusion, the present study developed and validated a novel comprehensive prediction model for PTC, which included, imprinted gene detection, components, microcalcification, and cytopathology. Internal and external validations demonstrated that the model had excellent predictive performance. The comprehensive prediction model can improve the diagnosis of PTC and reduce unnecessary operations. The visualized nomogram based on the prediction model was provided for clinical application. The new model is expected to solve the difficult problem of diagnosing PTC to a certain extent.

### Electronic supplementary material

Below is the link to the electronic supplementary material.

Supplementary Material 1

## Data Availability

The datasets used and analysed during the current study are available from the corresponding author on reasonable request.

## Abbreviations

TC: Thyroid carcinoma
PTC: Papillary thyroid carcinoma
FNA: Fine-needle aspiration
LOI: Loss of imprinting
QCIGISH: Quantitative Chromogenic Imprinted Gene In Situ Hybridization
FSE: Frozen section examination
AIC: Akaike information criterion
VIF: Variance inflation factor
LR: Likelihood ratio
DCA: Decision curve analysis
NRI: Net reclassification improvement
IDI: Integrated discrimination improvement
