# Supplementary material for: Imprinted gene detection effectively improves the diagnostic accuracy for papillary thyroid carcinoma
Source: BMC Cancer. 2024 Mar 20;24:359. doi: 10.1186/s12885-024-12032-z (PMC10953243; doi:10.1186/s12885-024-12032-z)

Supplementary Figure 1. Quantitative Chromogenic Imprinted Gene In Situ Hybridization (QCIGISH) thyroid nodule diagnostic model structure.


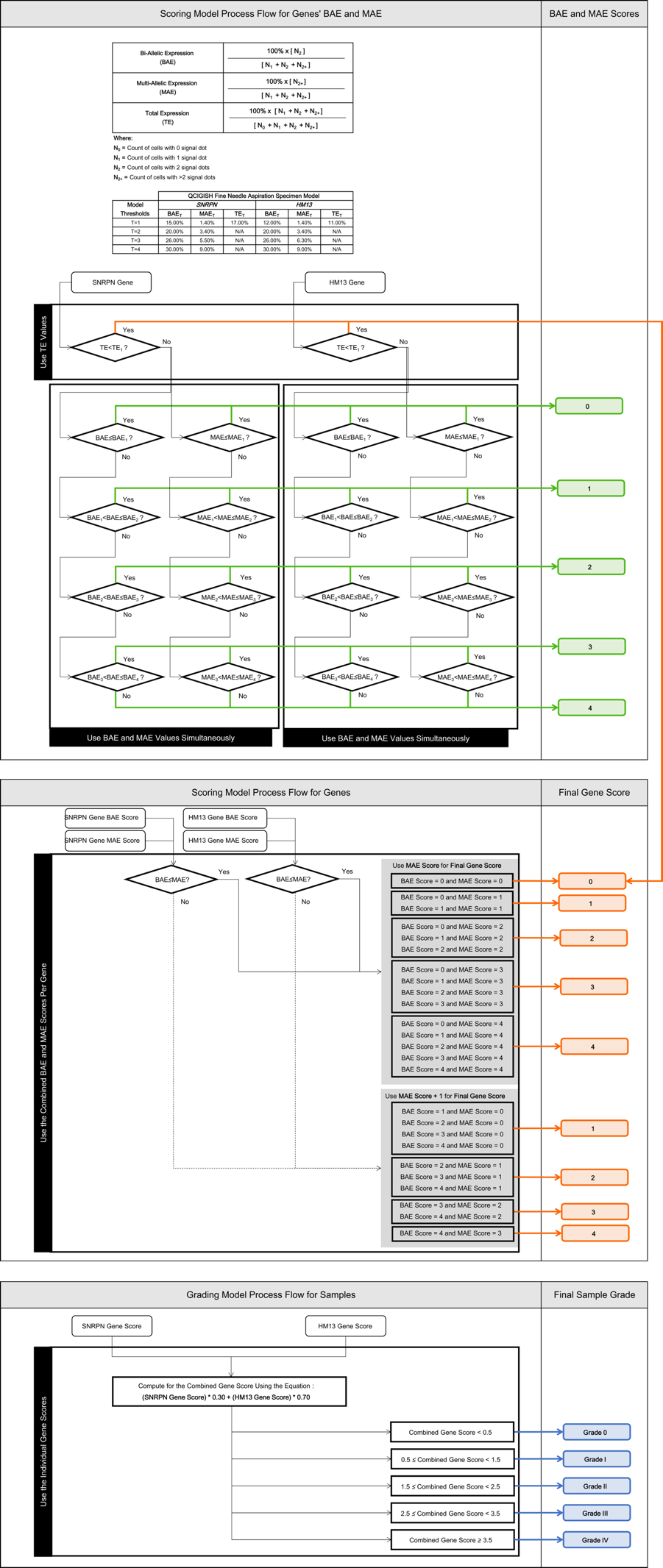


Supplementary Figure 2. The sensitivity analysis of the model. The ROC curve (a) and calibration plot (b) of the model in the re-created external validation cohort.


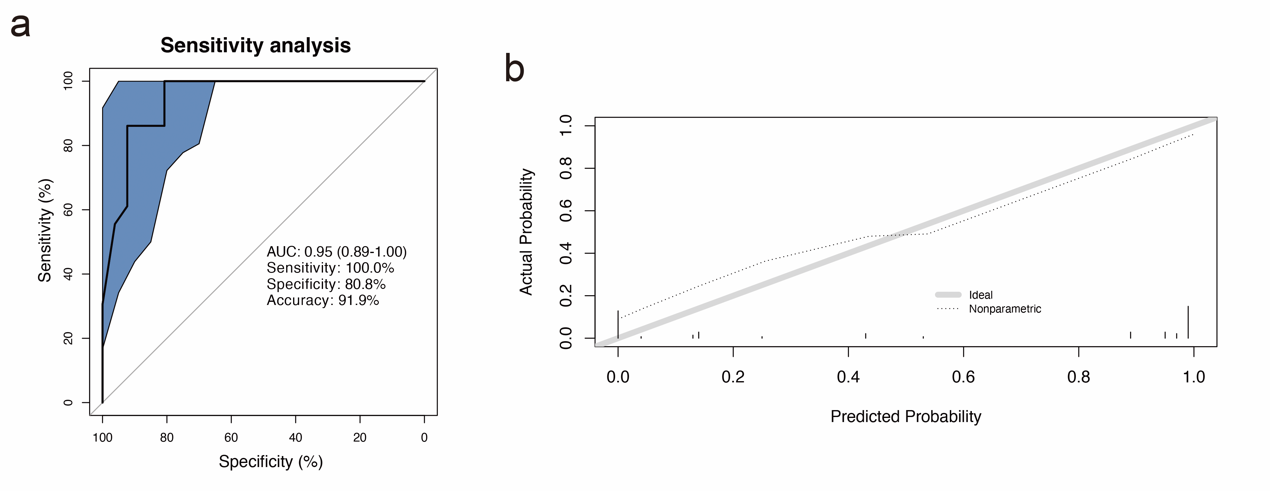

Supplement: Supplementary file 1 — Supplementary Material 1 [file 12885_2024_12032_MOESM1_ESM.docx]
